# Supplementary material for: Eggs of the mosquito Aedes aegypti survive desiccation by rewiring their polyamine and lipid metabolism
Source: PLoS Biol. 2023 Oct 24;21(10):e3002342. doi: 10.1371/journal.pbio.3002342 (PMC10597479; doi:10.1371/journal.pbio.3002342)
Supplement: Attachment — Submitted filename: 133348_1_rebuttal_2645582_rsp2t8.docx [file pbio.3002342.s010.docx]

**Manuscript number:** RC-2023-01827

**Corresponding author(s):**	Bhaskar, Bakthavachalu

Sunil, Laxman

*[Please use this template only if the submitted manuscript should be considered by the affiliate journal as a full revision in response to the points raised by the reviewers.*

*If you wish to submit a preliminary revision with a revision plan, please use our "[Revision Plan](https://docs.google.com/document/u/0/d/1HHp62Tfeb44nd3Gu_3ChB0b8gmMdEog7GjZcZFwHSiQ/edit)" template.* ***It is important to use the appropriate template to clearly inform the editors of your intentions.****]*

## 1. General Statements [optional]

*We thank the editor and the reviewers for their comments on this report. We have addressed all points in detail as best we can, and the resulting revision makes the results of our findings come out more clearly. A particular point coming out from our study is how much we can learn about molecular mechanisms of fundamental biological phenomena from what are effectively ‘non-genetic, non-model organisms’, such as the Aedes mosquito. We believe that this manuscript in its revised form not just makes observations about this general phenomenon (of cellular and organismal tolerance to loss of water), but also advances the field forward as well as immediately suggests multiple new directions to address new questions.*

## 2. Point-by-point description of the revisions

*This section is mandatory.* *Please insert a point-by-point reply describing the revisions that were already carried out and included in the transferred manuscript.*

**Response to reviewer comments**

**Reviewer #1: Major Points**

1) *I was hoping to see the gel run for various days of desiccation to support the conclusion that proteome remodeling occurs **during** the desiccation. Right now, the data in Fig. 2 come from a single day – 21 days post desiccation – so it still shows that proteomic remodeling happened during those 21 days but not exactly on which days.*

**Response:** Thank you for this suggestion. We have ourselves become quite interested in the exact nature and extent of proteome changes over time in this paradigm. Indeed, our findings in this study now open up so many future exciting directions including various possible molecular mechanisms that control this phenomenon. We are planning to carry out an extensive future study to compare the proteome of fresh and desiccated eggs quantitatively, and over time in order to explore these directions. At this stage though, a complete proteomic study is infeasible. However, our existing data still shows that *Aedes* eggs have acquired unique proteome – level changes which reiterates a distinct metabolic remodeling happening during the process of desiccation. We have added your point as an important consideration in the manuscript *(L207-209)*.

2) *In Fig 2B: Unclear what you’re using as a reference to say that “45 proteins increased and 125 proteins decreased in amounts” (L147-148). Relative to fresh eggs that were laid 48 hours ago? Why is this a good reference instead of say, fresh eggs that are 21 days old (same age as the desiccated eggs)?*

**Response:** Thank you for this comment, and this helps us to now clarify this point. Throughout the study, “fresh eggs” refer to eggs that were 48 hours old, maintained in moist conditions and not subjected to desiccation. Placing the eggs under moist conditions for 48 hours after egg laying was critical to allow embryonic development (Clements, 1992, ISBN: 9780412401800; Mundim-Pombo et al., 2021, [PMID: 34645492](https://pubmed.ncbi.nlm.nih.gov/34645492/)). By “desiccated eggs”, we mean fresh eggs (48 hours old) which are subsequently dried for a total period of 21 days by placing the eggs on Whatman filter paper. Therefore, the comparisons made in Figures 2 and 3 are between fresh eggs and desiccated eggs (21 days). Fresh eggs cannot be left for 21 days in moisture as they would hatch into larvae approximately between 48-72 hours after being laid (Clements, 1992; Mundim-Pombo et al., 2021, Rezende et al., 2008, [PMID: 18789161](https://pubmed.ncbi.nlm.nih.gov/18789161/)). Therefore, the only possible comparison is of fresh eggs at a stage where it would have acquired desiccation tolerance, with the fully desiccated 21-day old egg. We have added new content in the methods section *(L426-431)* on how eggs were collected for various experiments including the ones described in Figure 2 and also included a figure *(Figure S1D, L966-970)* to demonstrate the same.

3) *L90-L91: "...dried for up to 21 days" But the methods section states that the eggs were dried for 10 days on Whatman filter paper. The 21 days refers to the fact that the authors looked at eggs that were stored for 21 days after the 10 days of desiccation, no? Isn't that why the x-axis goes up to 21 days in Fig. 1C? Please clarify.*

**Response:** As mentioned in the previous comment response, “21 days” refers to 48 hours of embryonic development (which was achieved by leaving the eggs in moist conditions for 48 hours), followed by 21 days of desiccation on Whatman paper. These dried eggs were then rehydrated to check the percentage hatching *(Figure 1C)*. “21 days” does not mean 10 days of desiccation and 21 days of storage. We have accordingly modified the results section *(L98-102)*, the figure *(Figure 1A)*, its caption *(L802-809)* and the methods section *(L401-415)* to clarify and emphasize this point.

4) *Fig. 1C: related to above. What does "0-day post desiccation" mean in the x-axis? Is these 10 days of desiccation on Whatman paper + 0 days of storage? Similarly, what is 12 days or 21 days post desiccation on the x-axis? These are 10 + 12 days and 10 +21 days respectively?*

**Response:** “0 days post desiccation” refers to fresh eggs that are 48 hours old post egg laying and not subject to desiccation. This has been the control throughout our study. As mentioned above, it does not mean 10 days of desiccation and 0 days of storage. We have rephrased the the results section *(L98-102)*, the methods section *(L401-415)* and modified the illustration *(Figure 1A)* and its caption *(L802-809)* appropriately to describe how the desiccation assay was performed.

5) *Methods section on desiccation is very unclear (related to above). I cannot determine what the days in Fig. 1C means based on this methods section and the main text (and caption for fig. 1C).*

**Response:** We acknowledge the original methods had very brief statements, and so we have substantially revised the text *(L98-102)*, the methods section *(L401-415)* and improved the schematic *(Figure 1A)* and its caption *(L802-809)* to provide clarity on the experimental methods and on how the desiccation assays were designed. We have also included a separate section *(L426-431)* and an illustration *(Figure S1D, L966-970)* to demonstrate how samples were collected for various experiments.

6) *Fig. 2A: What are "D1" and "D2"? These are two trials of desiccation? For each lane (e.g., D1), did you combine 150 eggs and lysed them together for the single lane in the gel? Specify these points in the caption.*

**Response:** Yes, D1 and D2 refer to two independently conducted trials from desiccated eggs. 150 eggs were used in each trial, where these eggs were combined and lysed together for protein extraction in order to ensure sufficient material for both the qualitative visualization on SDS-PAGE gel and proteomic analysis. We have incorporated your suggestions in the revised methods section *(L443-447)* figure legends *(L841-845, 857-860)*.

7) *Related to above: Does the "21 day" correspond to 21 days **post** desiccation (i.e., "21" in the x-axis of Fig. 1C)? Or something else? Please specify in the figure caption.*

**Response:** Yes, 21 days in figure 1C corresponds to 21 days post-desiccation. We have clarified the definitions of fresh and desiccated eggs in response to comments 2, 3 and 4 above. To facilitate the understanding of how the desiccation assays were performed as well as the interpretations of Figure 1C, we have added text to the methods section *(L401-415)* and added explanations in the respective legends *(Figure 1A, L802-809)*.

8) *L145-146: What is the emPAI score? Give a one-sentence explanation.*

**Response:** The emPAI (exponentially modified protein abundance index) is an absolute quantitation method now widely used in proteomics, which allows comparisons of protein data acquired by LC-MS/MS (Ishihama et al., 2005, [PMID: 15958392](https://pubmed.ncbi.nlm.nih.gov/15958392/)). The PAI is the protein abundance index, and this is proportional to the logarithm of absolute protein concentration, and since detection relies on mass spectrometry, PAI will indicate the ratio of observed to observable (due to inherent peptide/ionization and detection properties) peptides. The emPAI values of proteins from one sample can now be compared with those in another sample, *especially* those obtained in contiguous MS runs using the exact same method, to determine increasing or decreasing proteins (as we have done in this study). Thank you for pointing this out and we have added the necessary information to the text *(L159-163)*.

**Reviewer #2**

1) *However, the method used to define the tolerance as anhydrobiosis, which forms the basis of this claim, is flawed. Physiological strategies for tolerating desiccation can be broadly divided into "desiccation avoidance," which involves maintaining the physiological state by preventing water loss, and "desiccation tolerance," which involves responding to water loss by changing the metabolic system. Desiccation tolerance can be further categorized into two types: hypometabolism, which reduces the metabolic rate, and ametabolism, which completely stops metabolism. The former is general desiccation tolerance, while the latter is defined as anhydrobiosis (Keilin, 1959. doi: 10.1098/rspb.1959.0013). To be classified as anhydrobiosis, the authors must demonstrate that metabolism, including respiration, has ceased completely, not merely that water content has been significantly reduced. The authors claim that the weight of Ae. aegypti eggs dropped by about 65% and that they were still able to hatch even after 21 days of desiccation, as evidence of anhydrobiosis. However, this only confirms the tolerance to desiccation exhibited by many insects living in arid regions.*

**Response:** We primarily see this study as a short discovery report that will open multiple directions of future inquiry in this space and we greatly appreciate these points. The responses below, therefore, are elaborate and considered, and we hope will clarify these points extensively.

We agree with the definition of anhydrobiosis as a phenomenon of the ability of some cells and animals to enter into a reversible state of suspended metabolism (ametabolism), and indeed we are admirers of the wonderful explanation of anhydrobiosis as explained by Keilin et al. in 1959. However, we would like to point out that even during such a state, the basal level of metabolism needed for cellular maintenance and repair has to exist (Bosch et al., 2021, [PMID: 34347349](https://pubmed.ncbi.nlm.nih.gov/34347349/); da Silva et al., 2019, [PMID: 30266630](https://pubmed.ncbi.nlm.nih.gov/30266630/); Garcia, 2011, [PMID: 22116292](https://pubmed.ncbi.nlm.nih.gov/22116292/); Pazos-Rojas et al., 2019, [PMID: 31323038](https://pubmed.ncbi.nlm.nih.gov/31323038/)). The definitions of ‘hypometabolism’ and ‘ametabolism’ were made mostly in the 1950s, when it was primarily possible to only obtain bulk estimates of respiration or glycolysis. Indeed, in every known example of desiccation tolerance or dormancy, there is a decrease in the energetic arms of glycolysis and the TCA cycle (da Silva et al., 2019; Dinakar & Bartels, 2013, [PMID: 24348488](https://pubmed.ncbi.nlm.nih.gov/22833170/); Erkut et al., 2013, 2016, [PMID: 24324795](https://pubmed.ncbi.nlm.nih.gov/24324795/), [PMID: 27090086](https://pubmed.ncbi.nlm.nih.gov/27090086/); Hibshman et al., 2020, [PMID: 33192606](https://pubmed.ncbi.nlm.nih.gov/33192606/); Ryabova et al., 2020, [PMID: 32723826](https://pubmed.ncbi.nlm.nih.gov/32723826/); Thorat & Nath, 2018, [PMID: 30622480](https://pubmed.ncbi.nlm.nih.gov/30622480/); Zhang et al., 2019, [PMID: 31019237](https://pubmed.ncbi.nlm.nih.gov/31019237/)). Entirely consistent with that, but with much more quantitative approaches that can make more complete inferences, our proteomics and metabolomics data (Figures 2 and 3), show a substantial decrease in glycolysis as well as the ATP and NADH producing arms of the TCA cycle (post α–ketoglutarate) clearly indicating a reduction in the key metabolic pathways involved in energy production *(L245-255, Figure 3A)*.

However, what we are now able to observe, is that there is a rewiring of the central carbon metabolism to support the production of protective molecules such as polyamines (in this case). This would be from a rerouting of flux, away from energy metabolism, but when that happens, it is essential that this ‘carbon and nitrogen’ is put somewhere. Energy-producing pathways during desiccation now are only active at very low efficiencies. The desiccated *Aedes aegypti* eggs are therefore indeed hypometabolic, and this conclusion is not made merely based on the fact that the total water content and weight of desiccated eggs has been reduced. Note that it is practically almost impossible to measure active respiration in desiccated *Aedes* eggs, since these measurements require a water-environment (and the entire purpose is lost if we add back water to the desiccated eggs). We will also point out that more recent studies by Erkut et al., 2013 in nematodes, which primarily relied on some proteomic measurements, as well as limited metabolite measurements, already hint that such phenomena occur in bona fide anhydrobiotes such as the pre-conditioned dauer larvae of *C. elegans*. By using approaches similar to those in our studies, we anticipate that there is a tremendous amount of new learning to be obtained in this area, and we will be able to better revise the definitions of desiccation tolerance or anhydrobiosis.

A ~65% loss in mass, while also considering the mass of the egg shell (and therefore the actual loss of mass in the embryo will be an even higher percentage) is substantial. While we have revised the text thoroughly to avoid the description of these embryos as ‘true anhydrobiotes’, we have rephrased this as desiccation tolerant, and hope readers will appropriately consider these aspects.

Finally, regarding the point of “*However, this only confirms the tolerance to desiccation exhibited by many insects living in arid regions*.”, we agree, and point out that little or nothing is known about the pathways or means by which many of such insects (including insects that are major causes of diseases in human, livestock or agriculture) survive under extremely dry environments and till date remain phenomenological. Our entire molecular understanding of desiccation tolerance comes from a handful of model organisms such as yeasts, nematodes, and some tardigrades. This study demonstrates the systematic analysis of desiccation tolerance and survival under rapidly changing environmental conditions in a non-model insect - the mosquito, which is already known to have globally diversified due to its ability to adapt behaviorally and physiologically to environmental fluctuations (Diniz et al., 2017, [PMID: 28651558](https://pubmed.ncbi.nlm.nih.gov/28651558/); Halsch et al., 2021, [PMID: 33431560](https://pubmed.ncbi.nlm.nih.gov/33431560/) ; Miller & Loaiza, 2015, [PMID: 25569303](https://pubmed.ncbi.nlm.nih.gov/25569303/)). Our study is a substantial advance in this regard, providing interesting insights into mechanisms of desiccation tolerance in mosquito eggs, a property which could in turn contribute to global expansion of this insect. We anticipate that this work will lay foundation to several studies to control the spread of *Aedes* mosquitoes.

Given the length of this report, we have chosen to avoid an extensive discussion section, and only briefly summarized this point *(L65-78, L222-235, L245-255, L281-284)*.

2) *The lack of significant accumulation of trehalose and the absence of accumulation of IDPs suggest that the tolerance in the dried eggs is not anhydrobiosis, which means that the manuscript is actually a study of the desiccation tolerance of Aedes aegypti eggs (not anhydrobiosis, nor is it extreme desiccation tolerance). The manuscript should, therefore, be renamed as "Aedes aegypti eggs use rewired polyamine and lipid metabolism to survive desiccation".*

**Response:** We agree with your suggestion of changing the title of the manuscript and hence have renamed it to “*Aedes aegypti* eggs use rewired polyamine and lipid metabolism to survive desiccation”.

However, we believe that referring to organisms as anhydrobiotes just because of its ability to exclusively accumulate trehalose or IDPs during the desiccated state would be inappropriate, and hinders advances in this space. Please allow us a systematic explanation of the same, below, in three parts: on anhydrobiosis, on trehalose synthesis, and on IDPs.

Anhydrobiosis or desiccation tolerance involves drastic physiological changes during the induction of anhydrobiosis, survival during the desiccated state and exiting this state upon rehydration (Bosch et al., 2021; Crowe, 2014, [PMID: 24548118](https://pubmed.ncbi.nlm.nih.gov/24548118/); Dinakar & Bartels, 2013; Pazos-Rojas et al., 2019; Rajeev et al., 2013, [PMID: 23739051](https://pubmed.ncbi.nlm.nih.gov/23739051/); Ryabova et al., 2020). Multiple processes enable the cell to deal with physical challenges – to protect proteins and membranes, and to maintain cellular integrity *(L49-59, L225-232)* . Different molecular processes can be used to attain this. The role of trehalose has been best characterized, at a molecular level primarily in yeasts and in nematodes (Erkut et al., 2016). Trehalose functions to protect the integrity of the membrane by forming glass-like structures as well as functions as a protein chaperone (Crowe et al., 1998, [PMID: 9558455](https://pubmed.ncbi.nlm.nih.gov/9558455/); Erkut et al., 2011, [PMID: 21782434](https://pubmed.ncbi.nlm.nih.gov/21782434/); Tapia & Koshland, 2014, [PMID: 25456447](https://pubmed.ncbi.nlm.nih.gov/25456447/)). In order for organisms to utilize trehalose, they must first accumulate it substantially, and this can only be done by shifting to very high rates of gluconeogenesis (Calahan et al., 2011, [PMID: 21840858](https://pubmed.ncbi.nlm.nih.gov/21840858/); Erkut et al., 2011, 2016; Tapia & Koshland, 2014). Two principles emerge from our own (Gupta et al., 2019, [PMID: 31259691](https://pubmed.ncbi.nlm.nih.gov/31259691/); Varahan et al., 2019, 2020, [PMID: 31241462](https://pubmed.ncbi.nlm.nih.gov/31241462/), [PMID: 32876564](https://pubmed.ncbi.nlm.nih.gov/32876564/); Varahan & Laxman, 2021, [PMID: 34849891](https://pubmed.ncbi.nlm.nih.gov/34849891/); Vengayil et al., 2019, [PMID: 31604822](https://pubmed.ncbi.nlm.nih.gov/31604822/)), and other studies that have tried to a build systems-level understanding of various ways by which flux towards trehalose can be increased. First, cells need to have carbon reserves that can reroute towards trehalose biosynthesis, either if glycolytic flux is reduced and/or if gluconeogenic flux is increased. Second, the presumption of ‘ametabolic states’ is incorrect, since there is a primary reduction of glycolysis and respiration, with a concurrent rerouting of flux towards trehalose accumulation. While this flux re-routing is possible (through various means), as has been observed in several desiccation tolerant organisms like yeasts and nematodes, all of these organisms were present in media/growth conditions where various carbon sources are abundant. Note that a key point of this study is to highlight that mosquito eggs are different in their natural environment – eggs are essentially a ‘closed system’, with limited inputs of nutrients, and when in fresh water (where eggs are typically laid), these are very poor carbon sources *(L79-85)*. Hence, rerouting of flux towards trehalose in such cases will be practically impossible.

Note that in this context, as a strategy to overcome desiccation stress, other organisms like tardigrades rarely accumulate trehalose but instead rely on intrinsically disordered proteins to survive desiccation (Boothby et al., 2017, [PMID: 28306513](https://pubmed.ncbi.nlm.nih.gov/28306513/); Hesgrove & Boothby, 2020, [PMID: 33148259](https://pubmed.ncbi.nlm.nih.gov/33148259/)). Tardigrades, unlike nematodes or yeast are present typically in carbon-poor, water environments. Therefore, when viewed in the context we have explained above, unless they have suitable carbon stores, tardigrades will also be unable to ramp up trehalose production easily during the desiccation process. Therefore, it makes entirely more sense that tardigrades do not rely on trehalose, but instead utilize IDPs in desiccation tolerance. Before this study, there was no clear or established role for IDPs. Note that the function of the IDPs is also to protect proteins from denaturation, much like what was later found for trehalose (Crowe et al., 1998; Tapia & Koshland, 2014).

An alternate way to achieve similar physical ends would be to utilize polyamines. Studies suggest a critical role for polyamines in desiccation tolerance in nematodes, separate from trehalose (Erkut et al., 2013). The ability of polyamines at higher concentrations to protect DNA/RNA, or phase transition into glass-like forms (much like trehalose and IDPs) is extremely well established (Miller-Fleming et al., 2015[, PMID: 26156863](https://pubmed.ncbi.nlm.nih.gov/26156863/); Saminathan et al., 2002, [PMID: 12202757](https://pubmed.ncbi.nlm.nih.gov/12202757/)). Therefore, our findings establishing a protective role for polyamines would be entirely consistent with interpretations made under these contexts *(Figure 3A, 3B, L281-293)*.

Finally, we clarify the idea of desiccation tolerance in *Aedes* eggs. We establish the following – after desiccation, the eggs have substantially low glucose/glycolytic metabolism, and TCA cycle metabolites. This is seen both at the proteome and metabolite levels *(Figure 2 and 3)*. In addition, we demonstrate substantially lower amounts of lipids, both in the desiccated state and after rehydration *(Figure 2D and 2E)*. Our study points towards a novel aspect of how metabolic rewiring not only supports protection during the desiccated state, but also ensures reactivation of metabolism upon return of favourable conditions. In the *Aedes* eggs, desiccation tolerance which involves survival and sustenance of the pharate larvae inside the dried egg as well as the exit from this dried state upon exposure to water, can logically be achieved only by repurposing internal acetyl-CoA reserves, which come from increased fatty acid breakdown to synthesize polyamines *(Figure 4E)*. The polyamines also confer protection from the consequences of desiccation together with other enzymatic antioxidants and molecular chaperones *(Figure 2C)*. Fatty acids are utilized by the pharate larvae for its energetic needs during the dormant state as well as to fuel recovery upon rehydration.

Conclusively, we find that desiccation tolerance can be achieved not just by accumulating trehalose or IDPs, but also because of additional relevant mechanisms that are biochemically possible. Thereby, this study adds up to our current knowledge and understanding of possible ways by which cells can achieve the same end of desiccation tolerance, and survival upon rehydration.

3) *This manuscript provides interesting insights from the perspective of a metabolomic analysis for clarifying the mechanism of the "general" desiccation tolerance, not anhydrobiosis, in dried Ae. aegypti eggs. For instance, the accumulation of polyamines might contribute to desiccation tolerance. The authors suggest a relationship between the accumulation of polyamines and hatchability based on the fact that the inhibition of metabolic pathways resulted in a decrease in hatchability and polyamines. However, conclusive evidence of a causal relationship is not available. It is possible that the inhibitors disrupted metabolic pathways other than the polyamine synthesis, leading to a significant decrease in hatchability.*

**Response:** We understand the reviewer’s point made here; however, we would like to elaborate a clarification on this point. In order to test the role of polyamines in desiccation tolerance in *Aedes* eggs, we inhibited the polyamine biosynthetic pathway using difluoromethylornithine (DFMO). While there may be some non-target effects of a drug, as is true for every inhibitor, our choices of inhibitors were very deliberate and carefully considered. DFMO is extensively used as an anticancer drug to specifically target ornithine decarboxylase, the first (and rate-controlling) step of polyamine biosynthesis (LoGiudice et al., 2018, [PMID: 29419804](https://pubmed.ncbi.nlm.nih.gov/29419804/)). Importantly, we made our inferences based on two sets of experiments with DFMO. First, we confirmed that DFMO reduces polyamine accumulation in desiccated eggs *(Figure S4B)*. Next, we observed significant reductions in the hatching of desiccated eggs that were obtained from mosquitoes fed with the inhibitor *(Figure 4A)*. This is consistent with a conclusion that the accumulation of polyamines is essential for desiccation tolerance. As critical controls, we included the hatching of fresh eggs obtained from mosquitoes fed with the inhibitor (in a concurrently conducted experiment, with the same sets of mosquitoes). These eggs showed a high percentage of hatching, which was very similar to the untreated controls *(Figure 4A, L309-313)*. This minimizes the possibility that DFMO could inhibit other metabolic pathways that led to reduced hatching. While acknowledging the limitations of pharmacology, our data collectively *(Figure 4A, S4B)* are consistent with the likelihood that eggs where polyamine biosynthesis is inhibited, are sensitive to desiccation. Since it is not easily feasible to knock-out ODC in mosquitoes, which are still non-model organisms, it is practically implausible to do experiments that are more conclusive. In fact, such experiments as shown in this study have never been performed in mosquitoes (or other similar insects) before, and we therefore believe both the findings, and the approach (of feeding adult female mosquitoes with inhibitors before egg laying) to be substantial advances. We entirely anticipate that these approaches will stimulate future studies in the many new directions this study opens up.

4) *The quantitative values of polyamines were shown only as relative values based on the values in fresh eggs of the control group. Absolute quantification of polyamines, particularly ornithine, putrescine, and spermidine, should be essential for this manuscript.*

**Response:** We thank the reviewer for raising an important point here. However, it is almost impossible to do such an experiment, since the concentrations of metabolites are usually calculated on the basis of total cellular volume of the extracted cells, normalized to the number of cells (Bennett et al., 2008, [PMID: 18714298](https://pubmed.ncbi.nlm.nih.gov/18714298/)). Calculating the volume of a single egg, particularly that of a desiccated egg because of its distorted shape is almost impossible. Hence, the steady state levels of all measured metabolites including polyamines, are represented as relative values calculated using the peak areas which corresponds to the amount of the particular metabolite present in the sample, where we normalize to egg numbers *(L878-879)*. We have provided the peak areas of all the measured metabolites in Table S4. Note that relative metabolite comparisons are a near-universally accepted approach to show fold-level increases or decreases and as a reference, we include this extensive methods paper where we and others discuss the different, appropriate ways of metabolite comparisons (Walvekar et al., 2018, [PMID: 30345389](https://pubmed.ncbi.nlm.nih.gov/30345389/)).

5) *The statistical analysis throughout the study raises concerns, as the sole significant difference test employed is the student’s t-test. While this test is suitable for comparing two groups, it cannot be used for making comparisons between three or more groups. For instance, in the experiment depicted in Figure 4, a comparison of fresh and dried eggs in the control and inhibitor treatment combination would entail comparing four groups. To address this, a two-way analysis of variance ought to be conducted, followed by a post-test such as Bonferroni's or Tukey's multiple comparison test.*

**Response:** Thank you for allowing us to clarify this. In the case of our studies, it would be inappropriate to use a two-way ANOVA, and correct to use the unpaired t-tests, because we do not make any comparisons between three or more groups, although visually it might have appeared that way in Figure 4. The data in Figures 4A, 4B S4B and S4C consists of 4 samples – control fresh eggs, control desiccated eggs, treated fresh eggs and treated desiccated eggs. However, comparisons are only made between two groups at a time. These would be control fresh eggs versus control desiccated eggs; OR treated fresh eggs versus treated desiccated eggs OR control desiccated eggs versus treated desiccated eggs i.e., the comparisons are only made for the appropriate two sets. For illustrating these comparisons in an easily readable way, the graphs are presented together. A two-way ANOVA can only be used when comparisons are made between more than two groups or to determine the effect of 2 variables on an outcome which is not applicable in our case. Therefore, only an unpaired test (eg. a student t-test) is appropriate, and we make absolutely no point about multiple comparisons. We’ve included a table below, purely as a reference point, where the same comparisons were made using Wilcoxon’s ranked tests, which is a non-parametric test that only infers information in the magnitudes and signs of the differences between paired observations. Note that there is no change in the conclusions, nor is there any issue with significance for the specific samples compared (in the main manuscript figures). In addition to this, we have added additional text in the figure legends *(L904-907, L918-921, L1015-1017, L1022-1025)* and also modified the graphs in all the figures for a clearer illustration of the comparison sets.

| **Figure No.** | **Comparisons** | **Measurement** | **p value (Wilcoxon's rank-sum test)** | **Significance** |
| --- | --- | --- | --- | --- |
| 4A | Control fresh eggs vs Control desiccated eggs | % hatching | 0.08143 | ns |
|  | Treated fresh eggs vs Treated desiccated eggs |  | 0.02857 | * |
|  | Control desiccated eggs vs Treated desiccated eggs |  | 0.02857 | * |
| 4B | Control fresh eggs vs Control desiccated eggs | % hatching | 0.05714 | ns |
|  | Treated fresh eggs vs Treated desiccated eggs |  | 0.02857 | * |
|  | Control desiccated eggs vs Treated desiccated eggs |  | 0.02857 | * |
| S4B (i) | Control fresh eggs vs Control desiccated eggs | Relative ornithine levels | 0.02107 | * |
|  | Treated fresh eggs vs Treated desiccated eggs |  | 0.05907 | ns |
|  | Control desiccated eggs vs Treated desiccated eggs |  | 0.0294 | * |
| S4B (ii) | Control fresh eggs vs Control desiccated eggs | Relative putriscene levels | 0.02107 | * |
|  | Treated fresh eggs vs Treated desiccated eggs |  | 0.8857 | ns |
|  | Control desiccated eggs vs Treated desiccated eggs |  | 0.02857 | * |
| S4B (iii) | Control fresh eggs vs Control desiccated eggs | Relative spermidine levels | 0.02107 | * |
|  | Treated fresh eggs vs Treated desiccated eggs |  | 0.05907 | ns |
|  | Control desiccated eggs vs Treated desiccated eggs |  | 0.0294 | * |
| S4C | Control fresh eggs vs Control desiccated eggs | Relative lipid levels | 0.02107 | * |
|  | Treated fresh eggs vs Treated desiccated eggs |  | 1 | ns |
|  | Control desiccated eggs vs Treated desiccated eggs |  | 0.02857 | * |

*p<0.05, **p<0.01, ***p<0.001, ns - no significant difference.
