# Supplementary material for: Eggs of the mosquito Aedes aegypti survive desiccation by rewiring their polyamine and lipid metabolism
Source: PLoS Biol. 2023 Oct 24;21(10):e3002342. doi: 10.1371/journal.pbio.3002342 (PMC10597479; doi:10.1371/journal.pbio.3002342)
Supplement: Attachment — Submitted filename: Response letter_AedesDesiccation.docx [file pbio.3002342.s012.docx]

To,

The editor

Plos Biology	21^st^ Aug 2023

We thank the editor and the additional reviewers for the added detailed feedback on our manuscript, post revision (where we had already addressed all concerns of reviewers 1 and 2 from review commons). We appreciate your recognition of the conceptual novelty (and difficulty) of this study, as well as the fresh insights it brings in this still obscure area of life in extreme conditions without water. In this second revision, we have carefully addressed all the additional points raised by reviewers 3 and 4, as well as the other minor points. This includes new experimental data that is included, as well as several important clarifications. Point by point responses are provided below, and the changes in the manuscript are also indicated. We also include a marked-up version of the revised text, for the convenience of the reviewers.

With these changes, we believe that our discoveries reported in this manuscript are further strengthened, and also has improved clarity. We hope to have satisfied all concerns, and hope for a quick response for this exciting discovery.

Thank you,

Sunil Laxman	Baskar Bakthavachalu

Detailed responses to reviewers:

**Reviewer #3**

1) *It is unclear why larval length is measured and how this would relate to development. The direct measure of larval development (how quickly L1 larvae develop into L2, L3, L4, Pupae and adults) demonstrated the same relationship, but the rationale for measuring length was not clear. The relationship between size of larvae and development can actually be inverse with smaller individuals emerging from larvae that have developed more quickly. Unless there is a reason to present this data, I would remove it as it is not clear what it tells us about development.*

**Response:** Thank you for this comment. In general, we agree with your point about the relationship between larval size and development. The primary purpose of including this data was to better characterize and compare larvae hatching from either desiccated or non-desiccated eggs (since there is no data on this that is publicly available). Our assessments in this regard are largely qualitative on the effect of desiccation on larvae. We have also included a more useful parameter of the duration that the larvae (hatching from fresh as well as desiccated eggs) take to eclose into an adult mosquito, as a table included in **Figure S1C**. We find that in both desiccated as well as fresh eggs, larvae take relatively similar times to develop. Using these collective observations, we find that desiccation does not overtly impair larval development post hatching. Additionally, we would like to reiterate that in Figure 1E we only address how similar the larvae are in terms of their size, irrespective of whether they hatch from fresh eggs or desiccated eggs. We do not make any claims or conclusions about the rates of development (for the exact reasons pointed out). We have accordingly modified the text in the results section ***(L147-152)*.**

2) *There is other work demonstrating the role of lipid metabolism in desiccation resistance during diapause. For example: Reynolds et al. 2012 Transcript profiling reveals mechanisms for lipid conservation during diapause in the mosquito, Aedes albopictus* [*https://doi.org/10.1016/j.jinsphys.2012.04.013*](https://doi.org/10.1016/j.jinsphys.2012.04.013)*. Urbanski et al. 2010 the molecular physiology of increased egg desiccation resistance during diapause in the invasive mosquito, Aedes albopictus Proc. R. Soc. B Biol. Sci., 277 (2010), pp. 2683-2692, -- These seem worth discussing in light of the protein data reported here.*

**Response:** These are both very interesting papers, and in fact can suggest some conserved mechanisms or principles that might be common to diapause and desiccation tolerance. Thank you for pointing this out. We have included these citations, and a short discussion in the manuscript, where we point to convergent features of diapause and desiccation, as well as distinguish between these phenomena. This is now included in line numbers ***(L68-71, L75-80, L401-417)*** of the manuscript. Given this convergence, our study suggests conserved principles of metabolic rewiring in these two phenomena. This allows a speculative hypothesis that the diapause state functions as a transition-point towards either recovery or extreme dormancy, determined by the extent and nature of metabolic rewiring.

3) *The inhibitor assays seem potentially problematic as interfering with lipid metabolism could have profound effects on female mosquito physiology including blood meal digestion and egg production. The study convincingly demonstrates the differences between treatments, but it seems these could be a subset of many differences in these eggs. Can the authors provide additional explanation or rationale for how this manipulation is targeting only the aspects of egg metabolism that they intend to manipulate?*

**Response:** We completely understand the reviewer’s point made here. We would like to clarify how and why we use this data, and how we interpret it. First, while drugs indeed can show off-target effects, our choice of inhibitors was made with exceptional care. In the case of both the drugs (DFMO and 2-BPA) used, it is possible that feeding female mosquitoes with the inhibitor may have implications on the physiology of the mosquito as well as its ability to lay eggs. For this reason, we used a concentration range based on published data (Chase et al., 1972; Batista et al., 2018; Koomoa et al., 2008) and titrated inhibitor dosage, until we found inhibitor concentrations where the fertility, fecundity and survival of the female mosquito were not notably affected. These details are included now in ***L326-329, L349-354, L581-587, L591-597***.

Specifically, to inhibit fatty acids we used 2-Bromopalmitate, which is perhaps one of best characterized, specific inhibitor of β–oxidation of fatty acids (Chase et al., 1972; Schulz, 1987). Additionally, we have included data regarding the specificity of action of the drug, by estimating total lipids in fresh and desiccated *Aedes* eggs under treated and untreated conditions (**Figure S4C**). Importantly, for mosquitoes treated with 2-BPA, the fresh eggs obtained from the same inhibitor-treated mosquitoes hatched normally (Figure 4B), and developed into normal larvae. This reiterates that ‘normal’ egg hatching and development is unaffected in mosquitoes. Only the hatching of desiccated eggs from mosquitoes treated with the same inhibitor substantially decreased (Figure 4B). Within the caveats of using any pharmacological inhibitor, this is about as specific and clean an experiment that can be possibly done. Our data indicates that the inhibitor affects egg lipid metabolism, particularly with respect to the process of desiccation. Due to the nature of the experimental set-up used, there will be some variability in the amount of blood ingested by a mosquito, as well as how much of the drug accumulates in the eggs. Despite that variability, we observe highly reproducible findings with respect to both the fresh eggs, and the desiccated eggs, only strengthening the conclusion that fatty acid oxidation is critical for the hatching and survival of desiccated eggs.

Finally, in the context of specific inhibition of lipid breakdown and its mechanistic role which we attribute to its essential requirement in restoring carbon homeostasis post-rehydration, the data from the rescue experiment shown in Figure 4C become compelling. In this experiment, we provide an alternate, otherwise unavailable carbon source (sucrose) to the rehydrating eggs that had been treated with the inhibitor, and this ~completely rescues hatching. This suggests that the inhibitor treatment was indeed specific to blocking lipid breakdown. It would be otherwise difficult to explain the near-complete rescue in hatching and survival that we can now observe.

Interestingly, the approach of feeding adult mosquitoes with a drug included in a blood meal, to inhibit metabolic pathways in the egg itself has not been used before (to our knowledge). We therefore anticipate that this approach, which we establish in this study, can become an invaluable approach that enables studies in the field of mosquito biology (or other insect pests with limited genetic tools available), with respect to studying the development of embryos within the egg.

4) *There needs to be an inclusion of the blood source (human? animal?) and appropriate ethical approvals for using blood included in the paper.*

**Response:** The mosquitoes were raised within a controlled iBSL2+ facility at inStem, Bangalore, which is dedicated to the growth of various mosquitoes, and uses human O+ve blood acquired commercially from a blood bank. Our studies adhered to the guidelines set forth by the human ethics as well as biosafety committees, and followed approved protocols for rearing the mosquitoes within this iBSL2 facility at inStem. Thank you for pointing this out, and we have included additional information in the methods section *(*included now in ***L433-441****)*.

**Reviewer #4**

1) *The major concern that I have is that there is not a proper control to disentangle aging and dehydration. The control is fresh eggs (two days old) and the dehydrated is 21 days of age. So, time and dehydration cannot be unlinked, making the conclusions linking dehydration and phenotypes observed as flawed in the current study. There are methods to allow the eggs to remain much more hydrated for extended periods (such as storage at high humidity), which would be a more appropriate control alongside the 48-hour sample. These processes that have been identified are likely involved in dehydration, but the results are far from solid without the extra control. Importantly, if we store Aedes aegypti in the lab, we will start seeing a reduction in viability of 40-60 days at conditions described from some lines, suggesting a very young to mid-life comparison.*

**Response:** We understand the reviewer’s concern. The establishment of a desiccation paradigm was done carefully, and in a controlled manner and some of this information was not clear in the previous version of the manuscript. We elaborate on how this was done below.

All experiments were carried out on synchronized eggs. Blood-fed females were first allowed to lay eggs in oviposition tubes for 1 hour in a dark chamber at 28°C, and synchronized eggs were collected. All synchronized eggs were maintained under moist conditions (on moist cotton) for 48 hours before they were subject to desiccation (**revised Figure 1A**, and **new Figure S1A**). This was specifically done in order to ensure that all the eggs used in our experiments are at the same developmental stage, and any differences that we observe between fresh and desiccated eggs at the protein or metabolite levels should predominantly not be due to variations in the states of development. In the study, the 48-hour-old eggs that are collected are termed “fresh eggs”. Note: It is impossible to keep eggs under moist conditions for longer than 48 hours, since after this stage they will hatch into 1^st^ instar larvae. After 48 hours of development, batches of fresh eggs were subjected to drying, by transferring them onto a filter paper, which will instantly remove ‘surface/external water, and allows systematic desiccation of the eggs for the required number of days (3, 6, 9, 12, 15 and 21). After this, the eggs are rehydrated to check the percentage hatching (**Figure S1A**). We have now revised **Figure 1A** and its legend (***L916-921***) for a clearer demonstration of how synchronized eggs were collected and desiccated, and included a new supplementary figure (**Figure S1A, *L1064-1073***) to illustrate the experimental set up of the desiccation assay.

In this revision, we also include new, additional controls that reiterate that fresh and desiccated eggs used throughout our experiments were at ~the same developmental stage. We clarified fresh and 21 days desiccated eggs and observed the embryos under a microscope. In both fresh and desiccated embryos, we can observe a well-defined head, thorax and similar segmentation patterns. These indicate that the embryos are at comparable stages of development (**new Figure S1B**). While there may be slight variances, accurately matching the exact states beyond this is unfeasible, due to the different experimental conditions necessary for examination. We have accordingly revised the text in the results and methods section to emphasize this point *(****L108-114, L123-129, L459-467****)*. We have included microscopic images of clarified fresh and 21 days desiccated eggs which shows the developmental states of the embryos (now as the new **Figure S1B, *L1074-1076***).

Our design of experiments with mosquito eggs came after many learnings from our earlier studies on the *dauer* larvae of *C. elegans*, which also tolerate desiccation. In that study, we had carried out substantial optimizations to ensure that the desiccation of the *dauer* larvae was done in a suitably controlled manner (Erkut et al., 2011; 2016). Learning from those studies, in this study, we believe we have established the most controlled and reproducible way to desiccate mosquito eggs, and anticipate that this approach will become the future standard.

2) *There is substantial literature about mosquito (and other insects) dehydration tolerance from eggs to adults, which has not been discussed or reviewed that needs to be included.*

**Response:** We do agree with this comment and have therefore included revised text regarding this content *(****L68-71, L75-80, L401-417****)*. Of note though, none of these prior studies ask the question from the perspective of the different needs cells have when enter the desiccation phase, and contrastingly exit this phase. The existing perspective therefore is that of how ‘desiccation protectants’ work, and these do not specifically address desiccation and restoration from the perspective of the metabolic state of a cell during desiccation. The data from our study now allow us to refine these distinct components, and provide insight into each aspect. We also uncover novel approaches to desiccation/restoration in the mosquito that still can be contextualized nicely within the framework of what is needed for desiccation. Importantly, we have been able to dissect processes leading to protection during desiccation, and revival during rehydration (and observe their metabolic connections). We therefore reiterate that our study provides a fresh, important, conceptual perspective on the fascinating phenomenon of desiccation tolerance.

**Additional information:**

We have now deposited the complete mass spectrometry proteomics data to PRIDE and the data is available via **ProteomeXchange with identifier PXD0445**25. The reviewer account credentials are as follows:

Username: [reviewer_pxd044525@ebi.ac.uk](mailto:reviewer_pxd044525@ebi.ac.uk)

Password: 29Izw2UJ
